# Upper limb modeling and motion extraction based on multi-space-fusion

**DOI:** 10.1038/s41598-023-36767-0

**Published:** 2023-09-26

**Authors:** Honggang Wang, Junlong Guo, Shuo Pei, Jiajia Wang, Yufeng Yao

**Affiliations:** 1 State Key Laboratory of Robotics and System, Harbin Institute of Technology, Harbin, 150001 China; 2 Tianzhi Institute of Innovation and Technology, Weihai, 264209 China

**Keywords:** Computational models, Data mining, Functional clustering, Bone, Stroke, Translational research, Applied mathematics

## Abstract

Modeling and motion extraction of human upper limbs are essential for interpreting the natural behavior of upper limb. Owing to the high degrees of freedom (DOF) and highly dynamic nature, existing upper limb modeling methods have limited applications. This study proposes a generic modeling and motion extraction method, named Primitive-Based triangular body segment method (P-BTBS), which follows the physiology of upper limbs, allows high accuracy of motion angles, and describes upper-limb motions with high accuracy. For utilizing the upper-limb modular motion model, the motion angles and bones can be selected as per the research topics (The generic nature of the study targets). Additionally, P-BTBS is suitable in most scenarios for estimating spatial coordinates (The generic nature of equipment and technology). Experiments in continuous motions with seven DOFs and upper-limb motion description validated the excellent performance and robustness of P-BTBS in extracting motion information and describing upper-limb motions, respectively. P-BTBS provides a new perspective and mathematical tool for human understanding and exploration of upper-limb motions, which theoretically supports upper-limb research.

## Introduction

In the past decade, the field of medical robotics has gained momentum and technology has matured^1^. However, more than one billion people worldwide are disabled, and the number is growing rapidly^2^. Among them, more than 80% of people disabled due to stroke suffer from upper-limb disability, and only 10% can regain partial mobility of the upper limb after treatment^3^. Limb motion is highly correlated with neuronal activity (neural plasticity)^4,5^, which is a potential mechanism for upper-limb motion recovery^6,7^. Based on this, upper-limb rehabilitation robotics has flourished, providing strong support for upper-limb motion recovery^8^. Upper-limb rehabilitation robots have the advantages and potential of compensating for motor deficits, motion enhancement, restoration of upper-limb functions, and rehabilitation task performance^9–12^.

Despite this, there is still conflict between patients with upper limb disabilities and those undergoing medical rehabilitation. The upper-limb rehabilitation equipment (exoskeleton^6,12,13^ and end-effector^12,14^) widely accepted mode of rehabilitation, drives or compensates for the motion of the affected limb, and provides intense rehabilitative training. However, the global outbreak of COVID-19 has severely hampered the rehabilitation of disabled people^2^ and has greatly reduced the efficiency of rehabilitation. Furthermore, moderate-to-severe patients cannot independently move the affected limb during the pre-rehabilitation period; they can only receive passive rehabilitation training. This model fails to play the key role of the healthy limb and is inefficient in rehabilitation.

The rehabilitation efficacy can be improved by organically integrating the upper-limb rehabilitation robot with the patient’s sense of autonomy. Research on human^7,15–17^ and marmoset^4^ brains has shown that self-directed actions can repair damaged neural circuits in the brain to some extent. Furthermore, observational learning leads to the activation of the mirror neural system in the observer’s brain^18^ (observed with fMRI^19^) and had been shown to improve upper-limb motor function^18,20^. Mirroring motions^21^ and observational learning are both initiated by the patient's brain; therefore, the rehabilitation effect can be reversed to act directly on the brain, thus increasing the rehabilitation efficacy. Therefore, extracting motion information from the upper limb and implementing it in the rehabilitation process can lead to optimal upper-limb rehabilitation effects.

Information regarding the motion of the human upper limb consists mainly of motion angles and holistic motion. For upper-limb rehabilitation motion, the holistic motion of the upper limb is of significant concern. The method of analyzing human upper-limb motion information can advance the research process of upper-limb motion recovery. The scope of the analysis should include the major human upper-limb motor bones: clavicle, humerus, forearm, and palm.

Numerous scholars have analyzed and explored upper-limb motion from different perspectives. Motion capture systems (e.g. VICON), which have become more popular in recent years and can provide accurate information on upper-limb motions, are generally utilized as experimental tools, and are not widely promoted because of their high cost and low interference resistance performance^22,23^. The Kinect depth camera^24^ acquired 3D spatial points from different parts of the upper limb and processed the data that can be utilized to build a remote system for assessing upper-limb motility^25^, determining upper-limb kinematic parameters (joint range of motion, displacement in the local coordinate system, joint smoothness, upper-limb length, etc.)^26,27^, solving upper-limb joint motion angles^28–30^, combining sliding mode control algorithms for human–machine interaction^31^, applying Kalman filtering techniques to fuse multiple Kinect data and track human motion^32^, etc. While Kinect’s human skeleton recognition technology offers the advantages of high flexibility, low cost, and non-invasiveness^33^, the number of human joint spatial points identified and extracted from the upper limb is very limited. Innovative approaches (multi-camera data fusion^32^ and inertial sensor compensation^34,35^) and wearable devices^36–38^ have been developed to improve the accuracy of Kinect depth camera data and measure upper-limb motion angles. However, the simplification of the upper limb to six degrees of freedom (DOFs) and below is inconsistent with the physiological properties of the upper limb. Meanwhile, a sizable research has been conducted for modeling upper-limb motion, including dissecting shoulder motion during the design of the upper-limb exoskeleton (ARMin series^39–41^, HARMONY^42^, CLEVERarm^43^, WINDER^44^, ChARMin^45^, etc.) and developing Denavit-Hartenberg based models of upper limb forward and reverse kinematics and dynamics^46,47^, hybrid twist-based model of shoulder kinematics^48^, rigid body model describing the kinematics of the scapula relative to the sternum^49^, and a musculoskeletal model of the upper limb^50–52^, etc. The established kinematic models describe localized motions of the upper limb and thus cannot completely describe the overall upper-limb motions.

**Figure 1:**
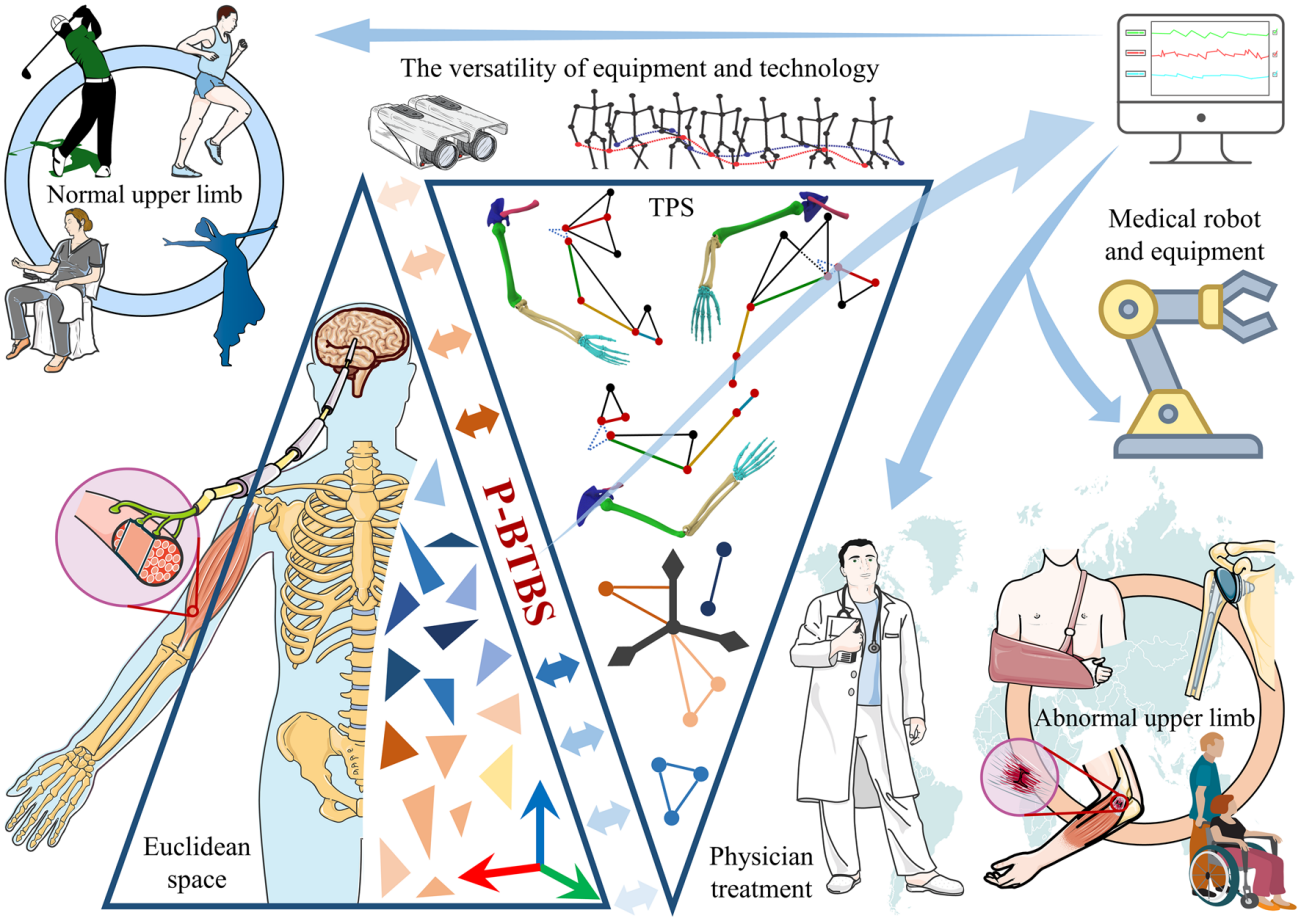
Overview of P-BTBS. The study targets of P-BTBS are the human upper limb (especially the abnormal upper limb, which is a global problem). The two dark blue triangles indicate the Euclidean space and Triangular primitive space (TPS), respectively. P-BTBS is aimed at dissecting upper-limb motions from the complex composition of the brain, nerves, muscles, and bones. The P-BTBS is suitable for various equipment and technologies (e.g. depth cameras and motion capture systems), and can be applied to a wide range of research areas (especially in the field of the medical robot).

In this study, we propose a general modelling and kinematic angle-solving method for simplified models of upper limbs with 1–8 DOFs (rotational) and named Primitive-Based triangular body segment method (P-BTBS). P-BTBS defines a triangular primitive space (TPS), maps the problem in Euclidean space to TPS, is utilized for inverse solving of 1–8 DOFs of the upper limb, and provides a mathematical description of upper-limb motions. The results largely satisfied realistic motion of the upper limbs. The coordinate information of a maximum of six spatial points and only one coordinate system transformation is required to analyze and extract the upper-limb motion information. In engineering applications, the Kinect’s human skeleton recognition technique is the optimal method for extracting 3D coordinates, and the six spatial points are largely consistent with those identified by this technique. Therefore, the P-BTBS (Fig. 1) has generic nature of the equipment, technology, and study targets. The ingenious and convenient modelling and calculation method is in line with the physiological characteristics of the upper limb, providing new ideas for upper-limb motion research and theoretical support for upper-limb mirroring rehabilitation^21,53,54^, motor function assessment^42,55,56^, upper-limb motion recognition^36,57^, etc. In conclusion, this study achieved ingenious and convenient kinematic analysis and description that better matched the physiological characteristics of the human upper limb by utilizing multi-spatial fusion, and provided theoretical support to advance the engineering application of upper limb modelling and motion extraction in various research fields.

## Results

### Sources of inspiration

Inspired by the fact that, defect-free Sierpiński triangles can be self-assembled on a silver surface in the microscopic world^58^, this led us to realize that matter moves with the property of triangles. Then we started to think about whether this was a base motion, which was the origin of the TPS.

### Simplified model of the upper limb

**Figure 2:**
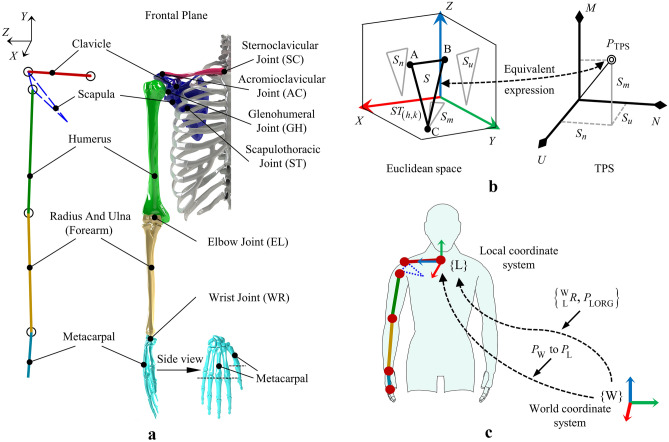
Overview of P-BTBS application object and method. (**a**) Four groups of bones and five joints (the scapulothoracic joint was not considered) are involved in this study. Each group of bones is given a distinctive color, and the body segments are represented by the corresponding color for the different bones. (**b**) A spatial triangle in Euclidean space consists of three spatial points with an area of *S*, the projected areas in the *X*–*Y*, *X–Z,* and *Y–Z* planes are *S*_*m*_, *S*_*n,*_ and *S*_*u*_, respectively. A spatial triangle in the triangular primitive space (TPS) is represented by a point whose coordinates are (*S*_*m*_, *S*_*n*_, *S*_*u*_). (**c**) The coordinates *P*_W_ in the world coordinate system can be expressed as *P*_L_ in the local coordinate system utilizing a coordinate system transformation (rotation $$\begin{array}{*{20}c} {\text{w}} \\ {\text{L}} \\ \end{array} R$$ and translation* P*_LORG_). Red points indicate actual spatial points on the joints (The locations are shown in Table 1).

As shown in Fig. 2a, the clavicle, scapula, humerus, forearm, and hand were the main components of the upper limb. The hand is composed of many bones including five metacarpals. The joint houses the main components of the upper-limb motion: the sternoclavicular joint (SC), elbow joint (EL), acromioclavicular joint (AC), glenohumeral joint (GH), scapulothoracic joint (ST), wrist joint (WR), and other joints in the palm. Both the EL and WR are composite joints.

To simulate the skeletal motions of the upper limb, the discontinuous line segments are divided, each of which is called the body segment (BS). In this article, the red, green, golden, and dark blue BSs indicate the clavicle, humerus, forearm, and metacarpal, respectively. The red, green, and golden BSs are equal to the length of the clavicle, humerus, and forearm, respectively, and the dark blue BS is equal to the length of the third metacarpal (longest metacarpal). The coordinate system was established using the International Society of Biomechanics (ISB) recommended coordinate system^59^. In numerous studies, scholars have mechanically simplified the joints of the upper limbs. The following simplifications were made in this study:

1. SC is a rotary joint^47,60^ with two DOFs rotating around the *X*- and *Y*-axes (the basal joint of the entire upper limb and serves as a mechanical support).
2. GH is a ball joint^47,61,62^ with three DOFs rotating around the *X*-,* Y*-*,* and *Z*-axes (the largest range of motion joint in the upper limb and greatly extending upper limb motion).
3. EL is a rotary joint^47,62^ with one DOF rotating around the *Z*-axes (the composite joint structure contributes significantly to the stability of forearm motion).
4. WR is a rotary joint^47,63^ with two DOFs rotating around the *X*- and *Z*-axes (the composite joint structure contributes significantly to the sophisticated motions and stability of the palm).
5. The clavicle is horizontal (parallel to the *X–Z* plane) when the arm is in its natural downward state.

The motions of the scapula are special among the limb bones because the motion of the joints connected to the scapula is somewhat complex (sliding and rotation of the SC; translation and rotation of the AC)^64^ and therefore cannot be conventionally simplified. The scapula has many muscles attached to it, which are used to maintain the stability of the shoulder joint and even the head and neck position and to provide strength for the motion of the upper limbs. Therefore, the most prominent role of the scapula should be reflected in biomechanics^49^, without denying that the scapula increases the flexibility of the upper limb in terms of kinematics. In addition, the scapula is located under the skin, which makes observing its motion in a non-invasive way difficult^65^, therefore the visual motion of the scapula was not considered in this study, but the upper-limb motion was observed directly by P-BTBS.

### Upper-limb modular motion model and coordinate system transformation

**Figure 3:**
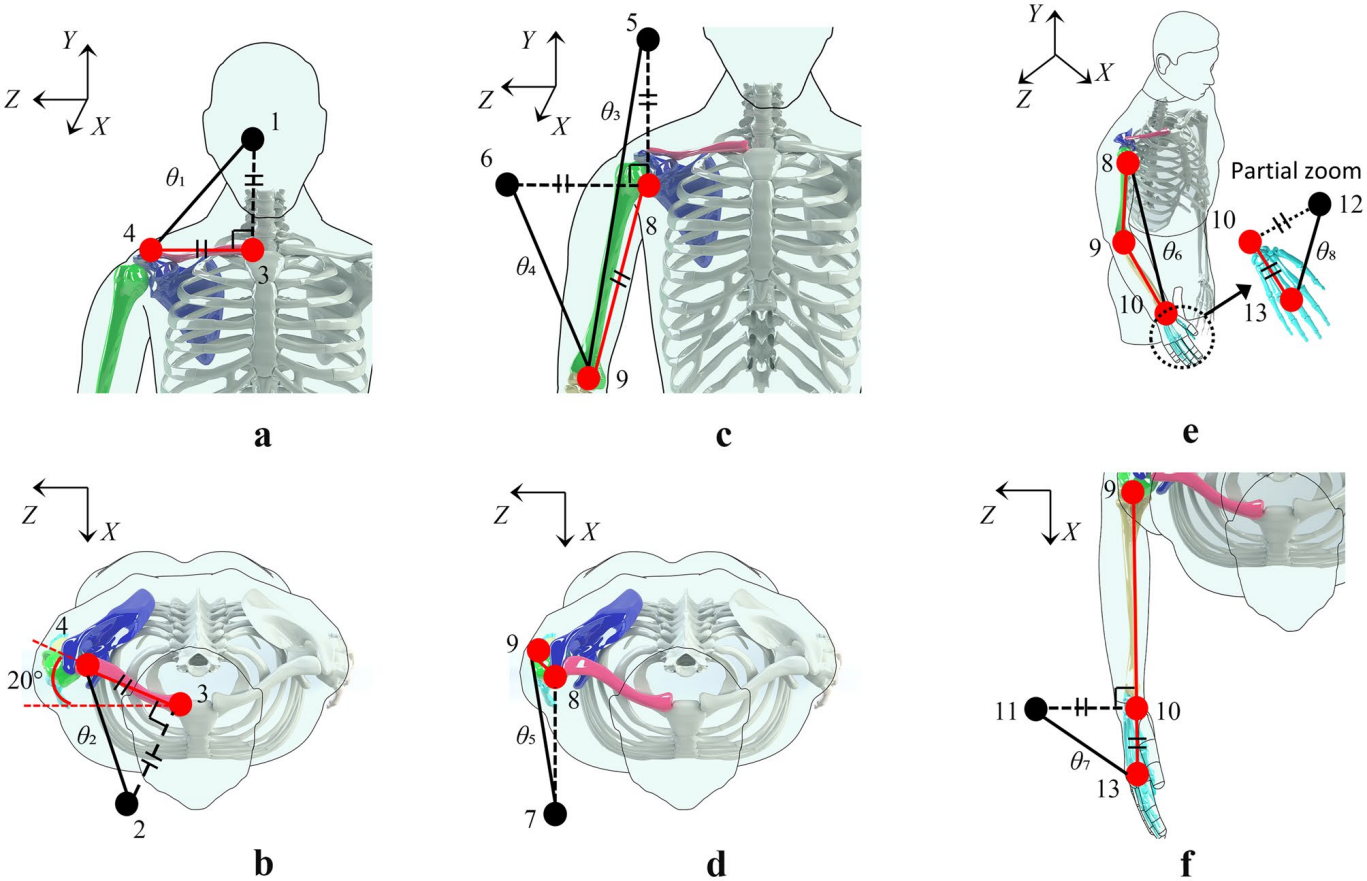
The definition of upper-limb modular motion model and the relationships between geometric features. Black points indicate virtual spatial points (calculated from actual spatial points), points 1, 2, and 3 are fixed points, and the remaining points are all moving points. Spatial point 3 is the origin of the local coordinate system. (**a**,**b**) Figures define the two spatial isosceles triangles of the clavicle and show the initial state of the natural dropping of the arms. (**c**,**d**) Figures define the three spatial isosceles triangles of the humerus, $l_{5,8} \bot l_{6,8} \bot l_{7,8}$. (**e**,**f**) Figures define the spatial triangles of the forearm and two spatial isosceles triangles of the metacarpal, $l_{9,10} \bot l_{10,11} \bot l_{10,12}$. The motion information represented by the spatial triangles is shown in Table 2.

The locations of all actual spatial points and bones are presented in Table 1. The length and spatial geometry relationships are shown in Fig. 3, and the angle between the clavicle and frontal plane was 20 degree^66^. The geometric features of the virtual spatial points can be obtained according to the definition of triangular spatial points, and the coordinates of the virtual spatial points are calculated using the coordinates of the actual spatial points. The spatial coordinates of *P*_1–2_ and *P*_5–7_ can be easily obtained as shown in Fig. 3. The spatial coordinates of *P*_11_ and *P*_12_ can be estimated using Eq. (1), which is obtained from the vertical and length relationships:

1 $$ \begin{array}{*{20}c} {\left[ {\begin{array}{*{20}c} {x_{9} - x_{10} } & {y_{9} - y_{10} } & {z_{9} - z_{10} } \\ {0} & 1 & 0 \\ {x_{i} - 2x_{10} } & {y_{i} - 2y_{10} } & {z_{i} - 2z_{10} } \\ \end{array} } \right]\left[ {\begin{array}{*{20}c} {x_{i} } \\ {y_{i} } \\ {z_{i} } \\ \end{array} } \right] = } {\left[ {\begin{array}{*{20}c} {x_{10} \left( {x_{9} - x_{10} } \right) + y_{10} \left( {y_{9} - y_{10} } \right) + z_{10} (z_{9} - z_{10} )} \\ {y_{10} {\text{ or }}x_{10} } \\ {l_{10,13}^{2} - x_{10}^{2} - y_{10}^{2} - z_{10}^{2} } \\ \end{array} } \right]} \\ \end{array} , $$

where *i* = 11 or 12, *l*_10,13_ denotes the length of vector $\overrightarrow {L}_{10,13}$.

**Table 1:** Definition of actual spatial points.

Actual spatial point	Location	Follow the motion
3	SC	Fixed point
4	AC	Clavicle
8	GH	Humerus
9	EL	Humerus
10	WR	Forearm
13	End of third metacarpal	Palm

Note that point 8 defines the motion of the humerus.

The local coordinate system was established using ISB’s suggested method, with the origin located at point 3. As shown in Fig. 2a,c rotation $$\begin{array}{*{20}c} {\text{w}} \\ {\text{L}} \\ \end{array} R$$ and translation *P*_LORG_ transformation relationship exists between local and world coordinate systems. Rotational transformations follow the form of *Z-Y-X* Euler angles. The coordinates of spatial point 3 in the world coordinate system are (*x*_3_, *y*_3_, *z*_3_) as a result of Eq. (2).

2 $$ P_{{{\text{LORG}}}} = \left[ {x_{3} ,y_{3} ,z_{3} } \right]. $$

The rotation matrix of Euler angles as shown in Eq. (3),

3 $$ \begin{array}{*{20}c} {\begin{array}{*{20}c} {\text{w}} \\ {\text{L}} \\ \end{array} R = \left[ {\begin{array}{*{20}c} {\text{c}\alpha \text{c}\beta } & {\text{c}\alpha \text{s}\beta \text{s}\gamma - \text{s}\alpha \text{c}\gamma } & {\text{c}\alpha \text{s}\beta \text{c}\gamma + \text{s}\alpha \text{s}\gamma } \\ {\text{s}\alpha \text{c}\beta } & {\text{s}\alpha \text{s}\beta \text{s}\gamma + \text{c}\alpha \text{c}\gamma } & {\text{s}\alpha \text{s}\beta \text{c}\gamma - \text{c}\alpha \text{s}\gamma } \\ { - \text{s}\beta } & {\text{c}\beta \text{s}\gamma } & {\text{c}\beta \text{c}\gamma } \\ \end{array} } \right],} \\ \end{array} $$

where c*x* = cos(*x*) and s*x* = sin(*x*); *α*, *β*, and *γ* denote the rotation angles around the *Z*, *Y*, and *X* axes, respectively.

In popular spatial position acquisition techniques (e.g. motion capture systems and depth cameras etc.) the posture of the world coordinate system can be adjusted. So, the singularity problem of Euler angles can therefore be mitigated by adjusting the position of the world coordinate system to avoid a 90-degree rotation of the intermediate axes.

As shown in Fig. 3a and b, the coordinates of spatial point 4 are known in the local coordinate system, as shown in Eq. (4),

4 $$ \begin{array}{*{20}c} {{}_{{}}^{{L}} P_{4} = \left[ { - \text{sin}20^\circ l_{3,4} ,0,\text{ cos}20^\circ l_{3,4} } \right]^{\text{T}} } \\ \end{array} $$

where *l*_3,4_ denotes the length of the vector $\overrightarrow {L}_{3,4}$.

The transformation relationship for coordinate point 4 is given by Eq. (5), where ${}_{{}}^{\text{W}} P_{4}$ are the coordinates of point 4 in the world coordinate system and acquired by motion capture system.

5 $$ \begin{array}{*{20}c} {{}_{{}}^{\text{W}} P_{4} = {}_{\text{L}}^{\text{W}} R{}_{{}}^{\text{L}} P_{4} + P_{{\text{LORG}}} } \\ \end{array} $$

Substituting Eqs. (2), (3) and (4) into Eq. (5), and a function of *α*, *β*, and *γ* can be determined. As shown in Fig. 6c and d, for the known world coordinate system *β* = 0°and *γ* = 90°. The value of *α* = 88.82° was determined using the Trust-Region-Dogleg algorithm.

Given the values of *α*, *β*, and *γ*, the local coordinates of all spatial points can be evaluated using Eq. (6).

6 $$ \begin{array}{*{20}c} {{}_{{}}^{\text{L}} P = {}_{\text{L}}^{\text{W}} R^{ - 1} \left( {{}_{{}}^{\text{W}} P - P_{{\text{LORG}}} } \right)} \\ \end{array} $$

### The Euclidean space

The triangular transformation relationship of the simplified upper limb model is presented in Table 2. The triangles can be selected according to the DOFs required to estimate the motion angles and describe the motion of the upper limb, which reflects the generality of the model. If the required DOFs are equal to *i*, there exist ${\text{C}}_{8}^{i}$ choices.

**Table 2:** BS characterization information.

The BS	The DOF	Actual spatial point	Virtual spatial point	The BS’s triangle
BS-1-scapula	2-*X/Y*	3 and 4	1 and 2	∆_1,3,4_–*X*–$\theta_{1}$, ∆_2,3,4_–*Y*–$\theta_{2}$
BS-2-humerus	3-*X/Y/Z*	8 and 9	5, 6 and 7	∆_5,8,9_–*Y*–$\theta_{3}$, ∆_6,8,9_–*Z*–$\theta_{4}$, ∆_7,8,9_–*X*–$\theta_{5}$
BS-3-forearm	1-EL	8, 9 and 10	–	∆_8,9,10_–EL–$\theta_{6}$
BS-4-metacarpal	2-*X/Z*	10 and 13	11 and 12	∆_10,11,13_–*X*–$\theta_{7}$, ∆_10,12,13_–*Z*–$\theta_{8}$

2-*X*/*Y* denotes two DOFs for rotation about the *X*- and *Y*-axes, ∆_1,3,4_-*X*-$\theta_{1}$ denotes ∆_1,3,4_ description of BS rotation about the *X*-axis, denoted by $\theta_{1}$. Other expressions were similar.

The motion angles of the upper limb were estimated using Eqs. (7), (8), (9), (10) and (11).

7 $$ \begin{array}{*{20}c} {\theta_{1} = \frac{\pi }{2} - \text{arccos}\left( {\frac{{y_{4} }}{{y_{1} }}} \right)} \\ \end{array} , $$

8 $$ \begin{array}{*{20}c} {\theta_{2} = \frac{\pi }{2} - \text{arccos}\left( {\frac{{x_{2} x_{4} + z_{2} z_{4} }}{{{\text{y}}_{1}^{2} }}} \right)} \\ \end{array} , $$

9 $$ \theta_{6} = \pi - \text{arccos}\left( {\frac{{l_{8,9}^{2} + l_{9,10}^{2} - l_{8,10}^{2} }}{{2l_{8,9} l_{9,10} }}} \right), $$

10 $$ \begin{array}{*{20}c} {\theta_{7} = \frac{\pi }{2} - \text{arccos}\left( {1 - \frac{{l_{11,13}^{2} }}{{2l_{10,13}^{2} }}} \right)} \\ \end{array} , $$

11 $$ \begin{array}{*{20}c} {\theta_{8} = \frac{\pi }{2} - \text{arccos}\left( {1 - \frac{{l_{12,13}^{2} }}{{2l_{10,13}^{2} }}} \right)} \\ \end{array} . $$

As shown in Fig. 3c and d, $\overrightarrow {L}_{5,8}$,$\overrightarrow {L}_{6,8}$, and $\overrightarrow {L}_{8,7}$ are the normal vectors of the *X–Z*, *X–Y*, and *Y–Z* planes, respectively.

The normal vectors of planes ∆_5,8,9_, ∆_6,8,9_, and ∆_7,8,9_ can be expressed using Eq. (12).

12 $$ \begin{array}{*{20}c} {{\vec{\mathbf{\mathcal{A}}}} = \overrightarrow {L}_{9,8} \times \overrightarrow {L}_{9,5} , {\vec{\mathbf{\mathcal{B}}}} = \overrightarrow {L}_{9,8} \times \overrightarrow {L}_{9,6} ,{\vec{\mathbf{\mathcal{C}}}} = \overrightarrow {L}_{9,8} \times \overrightarrow {L}_{9,7} } \\ \end{array} $$

Substituting the normal vectors ${\vec{\mathbf{\mathcal{A}}}},\,\,{\vec{\mathbf{\mathcal{B}}}}$, and ${\vec{\mathbf{\mathcal{C}}}}$ into Eq. (13) to estimate *θ*_3–5_:

13 $$ \left\{ \begin{gathered} \theta_{3} = {\text{arccos}}\left( {{{{\vec{\mathbf{\mathcal{A}}}}\vec {L}_{6,8} } \mathord{\left/ {\vphantom {{{\vec{\mathbf{\mathcal{A}}}}\vec {L}_{6,8} } {\left\| {{\vec{\mathbf{\mathcal{A}}}}} \right\|_{2} \left\| {\vec {L}_{6,8} } \right\|}}} \right. \kern-0pt} {\left\| {{\vec{\mathbf{\mathcal{A}}}}} \right\|_{2} \left\| {\vec {L}_{6,8} } \right\|}}_{2} } \right)\text{ (a)} \hfill \\ \begin{array}{*{20}c} {\theta_{4} = {\text{arccos}}\left( {{{{\vec{\mathbf{\mathcal{B}}}}\vec {L}_{8,7} } \mathord{\left/ {\vphantom {{{\vec{\mathbf{\mathcal{B}}}}\vec {L}_{8,7} } {\left\| {{\vec{\mathbf{\mathcal{B}}}}} \right\|_{2} \left\| {\vec {L}_{8,7} } \right\|}}} \right. \kern-0pt} {\left\| {{\vec{\mathbf{\mathcal{B}}}}} \right\|_{2} \left\| {\vec {L}_{8,7} } \right\|}}_{2} } \right)\text{ (b)}} \\ \end{array} \hfill \\ \begin{array}{*{20}c} {\theta_{5} = {\pi \mathord{\left/ {\vphantom {\pi 2}} \right. \kern-0pt} 2} - {\text{arccos}}\left( {{{{\vec{\mathbf{\mathcal{C}}}}\vec {L}_{5,8} } \mathord{\left/ {\vphantom {{{\vec{\mathbf{\mathcal{C}}}}\vec {L}_{5,8} } {\left\| {{\vec{\mathbf{\mathcal{C}}}}} \right\|_{2} \left\| {\vec {L}_{5,8} } \right\|}}} \right. \kern-0pt} {\left\| {{\vec{\mathbf{\mathcal{C}}}}} \right\|_{2} \left\| {\vec {L}_{5,8} } \right\|}}_{2} } \right)\text{ (c)}} \\ \end{array} \hfill \\ \end{gathered} \right.. $$

The calculation of Eqs. (7) to (13) involves only trigonometric functions, which improve the speed of computer response and increase the robustness of P-BTBS.

The upper limb was divided into four segments: clavicle, humerus, forearm, and metacarpal, the lengths and numbers of which are denoted as* L*_1_, *L*_2_, *L*_3_, and *L*_4_, respectively. One body segment BS-*h* (*h* = 1, 2, 3, 4) is described by *k* spatial triangles, and the spatial triangle number *k* ranges from 1 to 3. One spatial triangle, *ST*_(*h*,*k*)_, can be characterized using three spatial points (i.e. points *A*, *B*, and *C*). These three points were selected in a clockwise order. As shown in Fig. 2b, the area of *ST*_(*h*,*k*)_ is denoted as *S*_(*h*,*k*),_ and the projected areas in the *X–Y*, *X–Z*, and *Y–Z* planes are* S*_*m*_, *S*_*n*_, and* S*_*u*_, respectively, and can be calculated using Eq. (14),

14 $$ \begin{array}{*{20}c} {\left\{ {\begin{array}{*{20}c} {S_{m} = \frac{1}{2}\det \left( {ST_{{\left( {h,k} \right),m}} } \right) = \frac{1}{2}\det \left[ {\begin{array}{*{20}c} {x_{A} } & {y_{A} } & 1 \\ {x_{B} } & {y_{B} } & 1 \\ {x_{C} } & {y_{C} } & 1 \\ \end{array} } \right]} \\ {S_{n} = \frac{1}{2}\det \left( {ST_{{\left( {h,k} \right),n}} } \right) = \frac{1}{2}\det \left[ {\begin{array}{*{20}c} {x_{A} } & {z_{A} } & 1 \\ {x_{B} } & {z_{B} } & 1 \\ {x_{C} } & {z_{C} } & 1 \\ \end{array} } \right]} \\ {S_{u} = \frac{1}{2}\det \left( {ST_{{\left( {h,k} \right),u}} } \right) = \frac{1}{2}\det \left[ {\begin{array}{*{20}c} {y_{A} } & {z_{A} } & 1 \\ {y_{B} } & {z_{B} } & 1 \\ {y_{C} } & {z_{C} } & 1 \\ \end{array} } \right]} \\ \end{array} } \right.} \\ \end{array} , $$

where det(*ST*_*x*_) denotes the determinant of matrix *ST*_*x*_.

Area *S*_(*h*,*k*)_ can be estimated using the projected areas in Eq. (14), as shown in Eq. (15),

15 $$ \begin{array}{*{20}c} {\left\{ {\begin{array}{*{20}c} {S_{{\left( {h,k} \right)}} = S_{m} \vec{\user2{m}} + S_{n} \vec{\user2{n}} + S_{u} \vec{\user2{u}}} \\ {\left| {S_{{\left( {h,k} \right)}} } \right| = \frac{1}{2}\sqrt {\det \left( {ST_{{\left( {h,k} \right),m}}^{2} } \right) + \det \left( {ST_{{\left( {h,k} \right),n}}^{2} } \right) + \det \left( {ST_{{\left( {h,k} \right),u}}^{2} } \right)} } \\ \end{array} } \right.} \\ \end{array} , $$

where $\vec{\user2{m}}$, $\vec{\user2{n}}$, and $\vec{\user2{u}}$ are the unit normal vectors of the *X–Y–Z* coordinate system.

The area of the *h*-th BS can be defined as *S*_BS-*h*_, as shown in Eq. (16).

16 $$ \left\{ \begin{gathered} S_{{\text{BS} - h}} = \sum\limits_{i = 1}^{k} {S_{{\left( {h,i} \right)}} } \;\;k = 1,2,3 \hfill \\ \begin{array}{*{20}c} {\left| {S_{{\text{BS} - h}} } \right|} \\ \end{array} = \frac{1}{2}\left\{ \begin{gathered} \det \left[ {\left( {\sum\limits_{i = 1}^{k} {ST_{{\left( {h,i} \right),m}} } } \right)^{2} } \right] \hfill \\ + \det \left[ {\left( {\sum\limits_{i = 1}^{k} {ST_{{\left( {h,i} \right),n}} } } \right)^{2} } \right] \hfill \\ + \det \left[ {\left( {\sum\limits_{i = 1}^{k} {ST_{{\left( {h,i} \right),u}} } } \right)^{2} } \right] \hfill \\ \end{gathered} \right\}^{0.5} \hfill \\ \end{gathered} \right.. $$

The area of each BS can be estimated using the matrix sum of squares, according to Eq. (16), and matrix *E*_*h*_ was utilized to characterize the BS, as shown in Eq. (17),

17 $$ \begin{gathered} E_{h} = \left( {\sum\limits_{i = 1}^{k} {ST_{{\left( {h,i} \right),m}} } } \right)^{2} + \left( {\sum\limits_{i = 1}^{k} {ST_{{\left( {h,i} \right),n}} } } \right)^{2} + \left( {\sum\limits_{i = 1}^{k} {ST_{{\left( {h,i} \right),u}} } } \right)^{2} \\ = \left[ {\begin{array}{*{20}c} {f_{11} } & {f_{12} } & {2k\left( {{}^{\Sigma }P_{d} + \ldots + {}^{\Sigma }P_{f} } \right) + 3k^{2} } \\ {f_{21} } & {f_{22} } & {2k^{2} {}^{\Sigma }P_{i\,} + 3k^{2} } \\ {f_{31} } & {f_{32} } & {2k^{2} {}^{\Sigma }P_{j} + 3k^{2} } \\ \end{array} } \right], \\ \end{gathered} $$

where ${}^{\Sigma }P_{x}$ = *x*_*x*_ + *y*_*x*_ + *z*_*x*_. *P*_*i*_ and *P*_*j*_ are the actual spatial points of BS-*h*, *P*_*d*_, …, *P*_*f*_ are virtual spatial points of BS-*h*, and *f*_*i j*_ are functions of *x*, *y*, and *z*.

Vector $E_{h}^{\text{P}}$ denotes the third column of matrix *E*_*h*_, as shown in Eq. (18).

18 $$ \begin{array}{*{20}c} {E_{h}^{\text{P}} = \left[ {\begin{array}{*{20}c} {2k\left( {{}^{\Sigma }P_{d} + \ldots + {}^{\Sigma }P_{f} } \right) + 3k^{2} } \\ {2k^{2} {}^{\Sigma }P_{i\,} + 3k^{2} } \\ {2k^{2} {}^{\Sigma }P_{j} + 3k^{2} } \\ \end{array} } \right]} \\ \end{array} \text{.} $$

The body segment matrix *E*^O^ in Euclidean space can be determined using Eq. (19),

19 $$ \begin{array}{*{20}c} {E^{^\circ } = \left[ {\begin{array}{*{20}c} {E_{\text{1}}^{\text{P}} } & {E_{\text{2}}^{\text{P}} } & {E_{3}^{\text{P}} } & {E_{\text{4}}^{\text{P}} } \\ {L_{1} } & {L_{2} } & {L_{3} } & {L_{4} } \\ \end{array} } \right]} \\ \end{array} , $$

where *L*_*h*_ denotes the length of the *h*-th BS.

### Triangular primitive space (TPS)

As shown in Fig. 2b, the basic physical quantities in the coordinate system *M*–*N*–*U* in the TPS are the projected areas of the spatial triangle in the *X*–*Y*, *X*–*Z*, and *Y*–*Z* planes, respectively, as shown in Eq. (20). Therefore, a point* P*_TPS_ in the TPS is equivalent to a spatial triangle in Euclidean space.

20 $$ \begin{array}{*{20}c} {\left\{ {\begin{array}{*{20}c} {\left| M \right| = \left| {S_{m} } \right|} \\ {\left| N \right| = \left| {S_{n} } \right|} \\ {\left| U \right| = \left| {S_{u} } \right|} \\ \end{array} } \right.} \\ \end{array} . $$

The metric distance between point *P*_TPS_ (*M*_*i*_, *N*_*i*_, *U*_*i*_) and origin point *O*_*M*- *N*-*U*_ can be estimated using Eqs. (14) and (20), as shown in Eq. (21). Thus, according to Eqs. (20) and (21) and the physical significance of the point in the TPS, there is no singularity issue.

21 $$ \begin{array}{*{20}c} {\sqrt {\left( {M_{i}^{2} + N_{i}^{2} + U_{i}^{2} } \right)} = \frac{1}{2}\sqrt {\sum\limits_{j = m}^{u} {\det \left( {ST_{{\left( {h,k} \right),j}}^{2} } \right)} } } \\ \end{array} = \left| {S_{{\left( {h,k} \right)}} } \right|. $$

**Figure 4:**
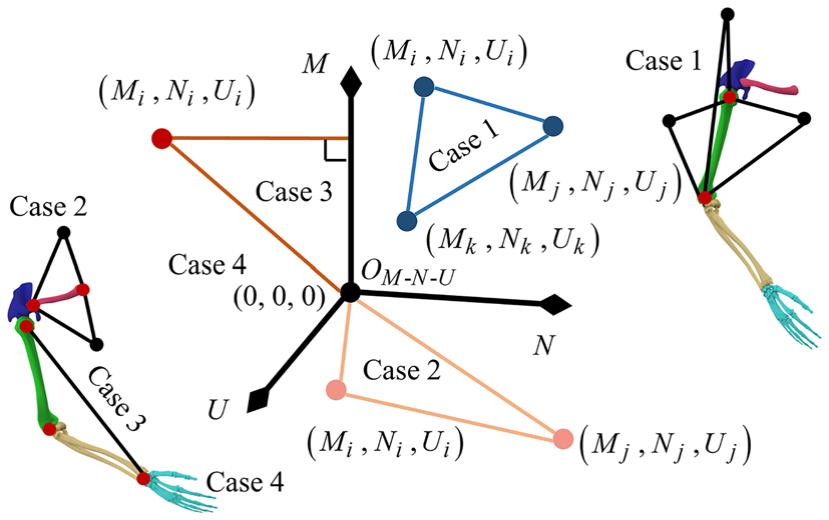
Four upper-limb modeling cases in TPS. The coordinates of the three points in case 4 are (0, 0, 0). The plane of case 3 consists of a point, a vertical point on the *M*-axis, and origin. The plane of case 2 consists of two points and the origin. The plane of case 1 consists of three points.

In TPS, a BS is expressed as three points, which has four cases, as shown in Fig. 4. Each case has three points, (*M*_*i*_, *N*_*i*_, *U*_*i*_), (*M*_*j*_, *N*_*j*_, *U*_*j*_), and (*M*_*k*_, *N*_*k*_, *U*_*k*_), where case 4 can be considered as three coincident points located at the origin of the coordinates.

A plane *S*_TPS_ in TPS can be represented as a matrix $S_{TPS}^{h}$ using three points, as shown in Eq. (22), and det($S_{TPS}^{h}$) = 0 is the equation for this plane.

22 $$ \begin{array}{*{20}c} {S_{TPS}^{h} = \left[ {\begin{array}{*{20}c} {M - M_{j} } & {N - N_{j} } & {U - U_{j} } \\ {M_{i} - M_{j} } & {N_{i} - N_{j} } & {U_{i} - U_{j} } \\ {M_{k} - M_{j} } & {N_{k} - N_{j} } & {U_{k} - U_{j} } \\ \end{array} } \right],} \\ \end{array} $$

where *h* = 1, 2, 3, 4.

The body segment matrix *E*^T^ in the TPS can be determined using the planes of the four body segments, as shown in Eq. (23).

23 $$ \begin{array}{*{20}c} {E^{\text{T}} = \left[ {\begin{array}{*{20}c} {S_{TPS}^{1} } & {S_{TPS}^{2} } \\ {S_{TPS}^{3} } & {S_{TPS}^{4} } \\ \end{array} } \right],} \\ \end{array} $$

### Action representation and inverse solving

The matrices representing the holistic motion of the upper limb, named the upper-limb body segment matrices, are obtained from the Euclidean space and TPS, respectively, as shown in Eqs. (19) and (23), respectively.

**Figure 5:**
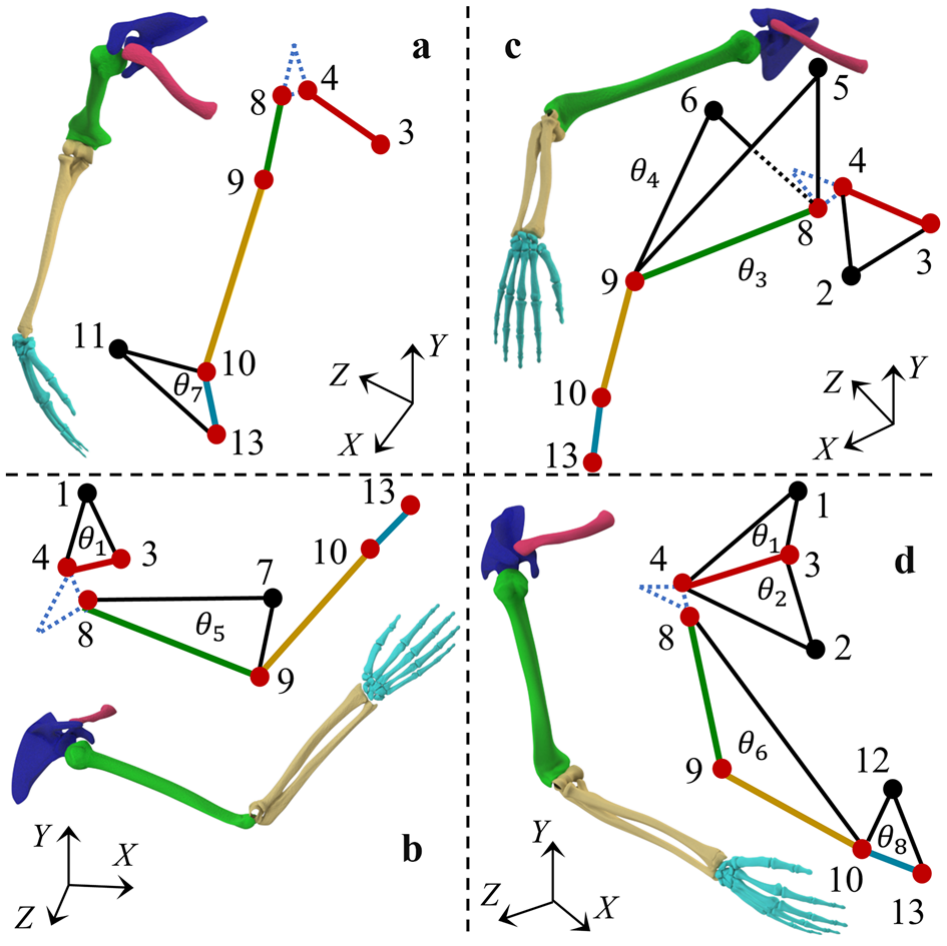
Diagram of various motions of upper limb and the corresponding spatial triangles. Arbitrary selection of spatial triangles according to the required DOFs. (**a**) Attention to just one DOF of motion of the hand. (**b**) Attention to one DOF of clavicle and humerus each. (**c**) Attention to one DOF of the clavicle and two DOFs of the humerus. (**d**) Focus on two DOFs of the clavicle, one DOF of the forearm, and one DOF of the palm.

In scientific research and engineering applications, attention has been paid to investigating the upper-limb motion of different bones with diverse rotational DOFs according to various research scenarios and engineering needs. P-BTBS was used to build spatial triangles to estimate the rotation DOFs of bones of interest. A spatial triangle represents the spatial state of the corresponding bone, as shown in Fig. 5.

The coordinates of the points in Fig. 4 were first determined by solving matrices *E*^T^ and $S_{TPS}^{h}$, then substituted into Eq. (21) to estimate the area, |*S*_(*h,k*)_|, of the corresponding spatial triangle. Obtaining *L*_*h*_ using the matrix* E*^O^ is straightforward.

Substituting* L*_*h*_ and |*S*_(*h,k*)_| into Eq. (24) to estimate the motion angle of the BS-1-Scapula, BS-3-Forearm, and BS-4-Metacarpal.

24 $$ \begin{array}{*{20}c} {\left| {S_{{\left( {h,k} \right)}} } \right| = \left\{ \begin{gathered} 1/2L_{h}^{2} \sin \left( {\theta_{i} } \right),h = 1,4 \hfill \\ 1/2L_{h - 1} L_{h} \sin \left( {\theta_{i} } \right),h\text{ = 3} \hfill \\ \end{gathered} \right.} \\ \end{array} . $$

*θ*_3-5_ for BS-2-Humerus can be estimated by solving Eq. (25), which was optimized using Eq. (13),

25 $$ \begin{array}{*{20}c} {\left\{ \begin{gathered} \cos \theta_{3} = \frac{{x_{9} - x_{8} }}{{\sqrt {\left( {z_{8} - z_{9} } \right)^{2} + \left( {x_{8} - x_{9} } \right)^{2} } }} = \frac{{ - \psi_{1} }}{{\sqrt {\psi_{3}^{2} + \psi_{1}^{2} } }} \hfill \\ \begin{array}{*{20}c} {\cos \theta_{4} = \frac{{y_{8} - y_{9} }}{{\sqrt {\left( {y_{8} - y_{9} } \right)^{2} + \left( {x_{8} - x_{9} } \right)^{2} } }} = \frac{{\psi_{2} }}{{\sqrt {\psi_{2}^{2} + \psi_{1}^{2} } }}} \\ \end{array} \hfill \\ \begin{array}{*{20}c} {\cos \theta_{5} = \frac{{z_{8} - z_{9} }}{{\sqrt {\left( {y_{8} - y_{9} } \right)^{2} + \left( {z_{8} - z_{9} } \right)^{2} } }} = \frac{{\psi_{3} }}{{\sqrt {\psi_{2}^{2} + \psi_{3}^{2} } }}} \\ \end{array} \hfill \\ \end{gathered} \right.} \\ \end{array} , $$

where *ψ*_1_ = *x*_8_–*x*_9_, *ψ*_2_ = *y*_8_–*y*_9_, *ψ*_3_ = *z*_8_–*z*_9_.

The coordinates of BS-2-Humerus in the TPS can be calculated using *E*^T^ with *h* = 2, *i* = 3, *j* = 4, and *k* = 5 by solving Eq. (22). Substituting the coordinates of BS-2-Humerus and *L*_2_ into Eq. (26) to estimate *ψ*_1–3_.

26 $$ \left\{ \begin{gathered} \psi_{1} = \frac{{ - \det \left( {ST_{{\left( {2,3} \right),m}} } \right)}}{{L_{2} }} = \frac{{ - 2M_{3} }}{{L_{2} }} \hfill \\ \psi_{2} = \frac{{ - \det \left( {ST_{{\left( {2,4} \right),u}} } \right)}}{{L_{2} }} = \frac{{ - 2U_{4} }}{{L_{2} }} \hfill \\ \psi_{3} = \frac{{\det \left( {ST_{{\left( {2,5} \right),n}} } \right)}}{{L_{2} }} = \frac{{2N_{5} }}{{L_{2} }} \hfill \\ \end{gathered} \right.. $$

The motion angles *θ*_3–5_ can finally be estimated by substituting Eq. (26) into Eq. (25).

### Simplified model of humeral motion

**Figure 6:**
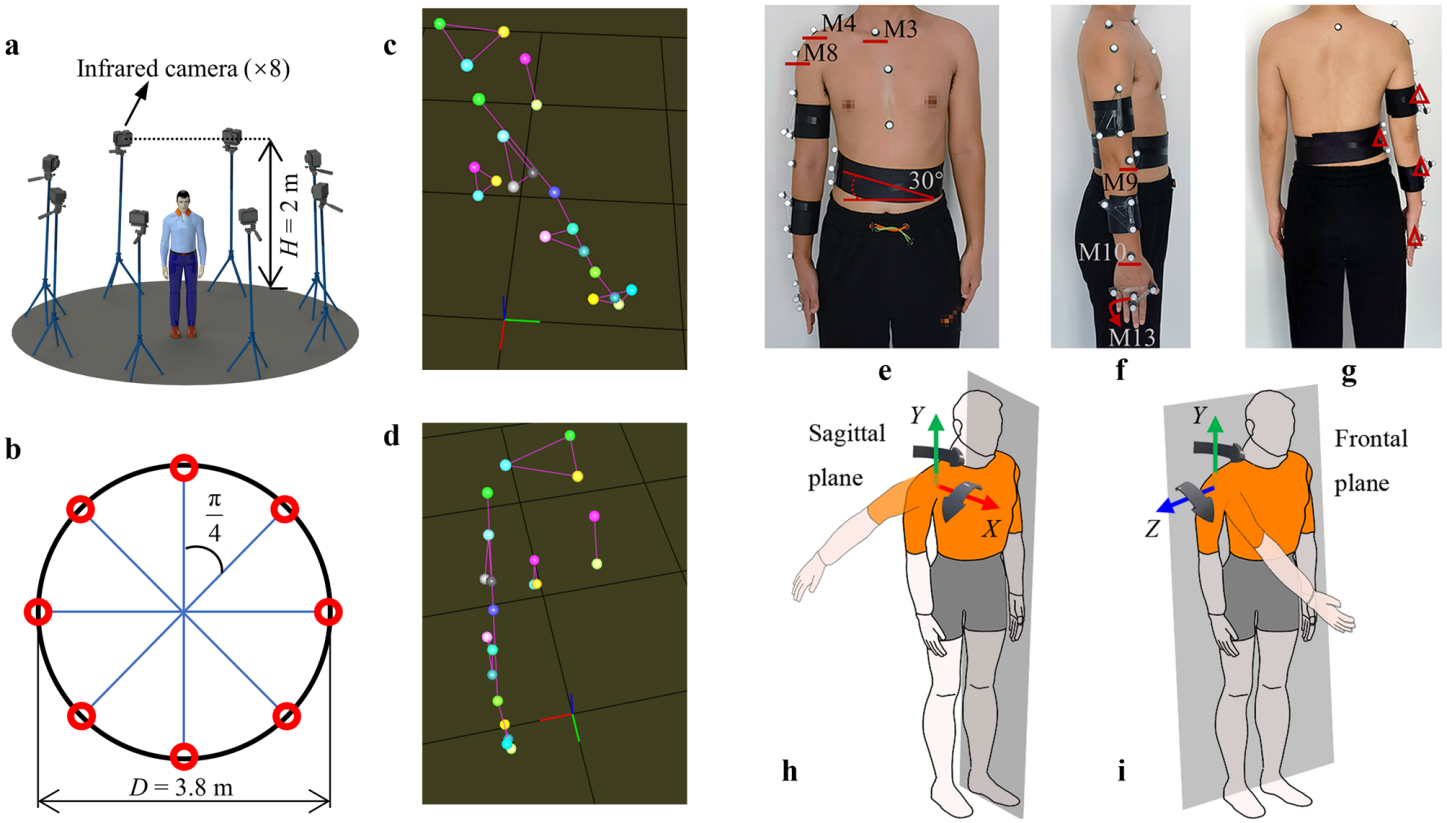
Experimental overview and humerus-motion simplified model. Motion capture lens (**a**) height and (**b**) layout. (**c**) Position and (**d**) posture of the world coordinate system. The red, green, and blue axes are the *X*, *Y*, and *Z* axes, respectively. The *X–Y* plane is parallel to the ground, and the local coordinate system is rotated 0° and 90° around the *Y* and *X* axes of the world coordinate system, respectively. (**e**–**g**) The six points labelled in the diagram are required for the P-BTBS, the remaining points are necessary for the motion capture system to calculate the exact values. The red triangle symbols show the placement of the triangular rulers. (**h**) and (**i**) The sagittal plane is parallel to the X–Y plane. The frontal plane is parallel to the Y–Z plane. The origin of the coordinate system is located at spatial point 3.

Humeral motions can be divided into two categories when the initial posture of the arms is in a natural downward state. One category is the combined motion of the *X* and *Y* axes, where the *X*–*Y* plane is parallel to the sagittal plane, as shown in Fig. 6h. When the initial motion of the humerus tends to move away from the sagittal plane, humerus posture can be decomposed using rotational DOFs around the *X* and *Y* axes. This decomposition results from the fact that two DOFs in the *X* and *Y* axes can determine any position of the humeral end in front of the sagittal plane.

The other is the combined motion of the *Y* and *Z* axes, and the *Y*–*Z* plane is parallel to the frontal plane, as shown in Fig. 6i. When the initial motion of the humerus moves away from the frontal plane, humerus posture can be decomposed using rotational DOFs around the *Y* and *Z* axes. This decomposition results from the fact that two DOFs in the *Y* and *Z* axes can determine any position of the humeral end in front of the coronal plane.

Determining the motion tendency of the humerus during the initial stages is important. Moving from the initial state, the motion category can be determined by comparing the magnitudes of *θ*_4_ and *θ*_5_. For *θ*_4_ < *θ*_5_, the motion belongs to the first category. Otherwise, motion belongs to the second category.

### Joint angle estimation during continuous motion

For healthy individuals, continuous motion is one of the most important motions in daily life. Continuous motion is a crucial rehabilitation aim for people undergoing upper-limb motion recovery.

To verify the performance of P-BTBS in solving joint angles during continuous upper-limb motion, motion capture experiments for seven DOFs upper limbs were performed. The experimental setup is illustrated in Fig. 6a and b, in which eight motion capture cameras were evenly distributed around the circumference. The circumference, height, and frame rate were approximately 3.8 m, 2 m, and 60 Hz, respectively. The participants stood at the center of the circumference. And the experimental procedure is shown in Supplementary Video 1.

The participants were in a good physical condition, without upper-limb injuries suffered within a month, and without strenuous exercise performed within a week. The skin near the marker point was first washed with medicinal alcohol before the experiment. The black bandages were then wrapped around key positions to minimize the effect of skin motion, as shown in Fig. 6e–g. Moreover, the black bandage was wrapped around the abdominal area to reduce the influence of the vertical motion of abdominal fat. The inclination angle was approximately 30°. The motion of a rigid body can be captured using a motion capture system with the highest accuracy. To obtain the exact values of the joint angles, several triangular rules were pasted onto the body as rigid bodies.

**Figure 7:**
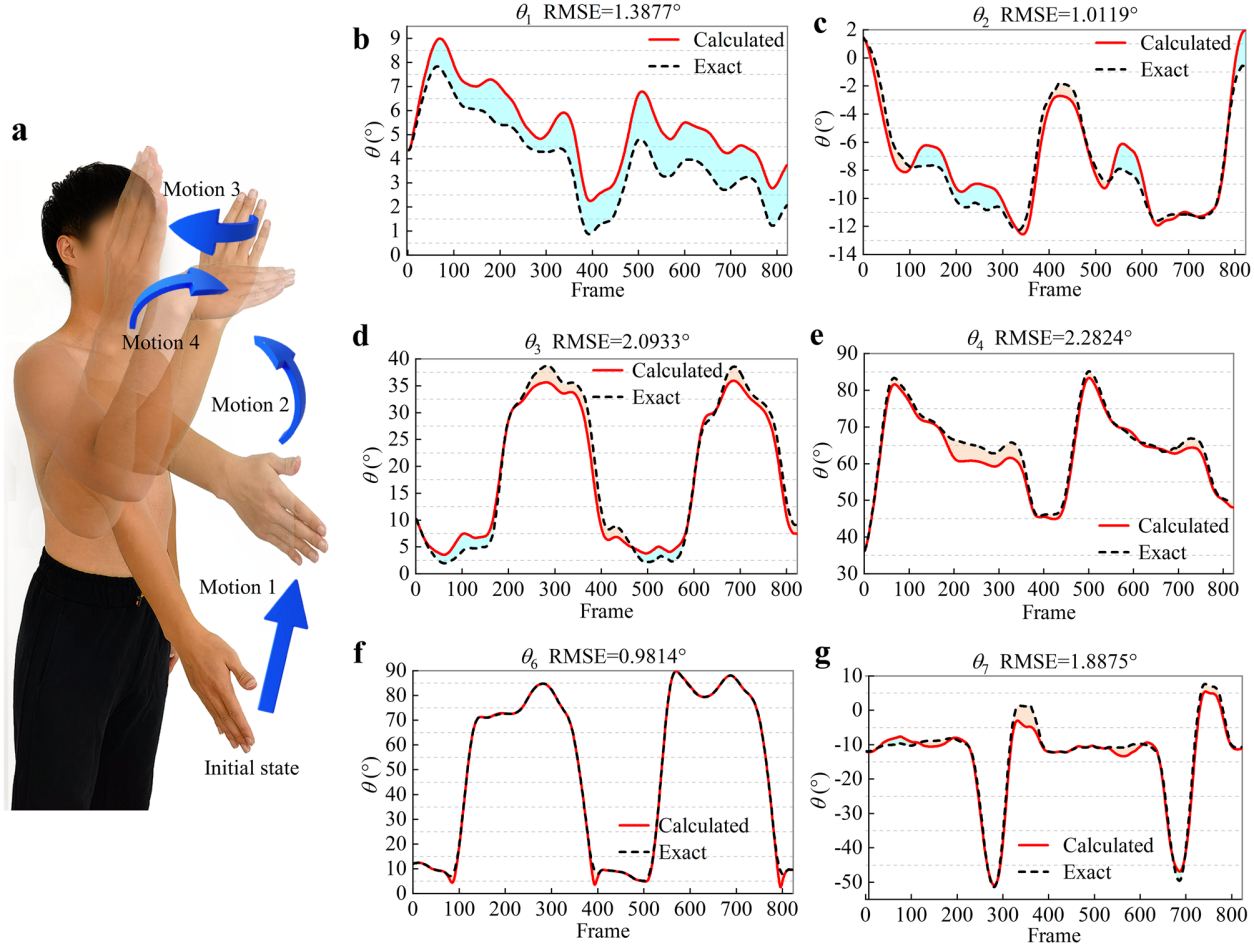
The experiment of upper-limb motion information extraction during continuous motion. The calculated and exact values are the results of the P-BTBS and motion capture system, respectively. The positive and negative results of subtracting the calculated value from the exact value are filled in the graph in light blue and light orange, respectively. The total error for each group of results is expressed as Root Mean Squared Error (RMSE). (**a**) Five consecutive upper-limb motions, repeated twice. The initial state is not in the natural downward state to avoid the upper limb obscuring the marker point during the initial. Action 1: a forward lift of the humerus. Action 2: a bend of the elbow. Action 3: a turn of the humerus around the *Y* axis. Action 4: an internal wrist snap, finishing with a return to the initial position and proceeding to the next motion. (**b**,**c**) Two angles of rotational motion of the clavicle. (**d**,**e**). The two angles of rotational motion of the humerus. As shown in the simplified model of humeral motion, the angle *θ*_5_ has no contribution to the action before the frontal plane, so is not calculated and measured. (**f**,**g**). The angle of rotation motion of the forearm and palm.

The P-BTBS is a general method for eight DOFs. To demonstrate the generality and advantage of handling highly redundant upper-limb motions, seven DOFs of the upper limb were chosen as the experimental target. Six upper-limb spatial points (M3, M4, M8, M9, M10, and M13) were selected, as shown in Fig. 6e–g. Seven spatial triangles were built, as shown in Fig. 5. The experimental motions are shown in Fig. 7a, where the initial state was not in naturally downward. The initial state was set up to avoid obscuring the side-marker points of the abdomen. The simplified model of humeral motion evidently shows that the experimental motions belong to the second category.

The experimental motions first started with the initial state and were lifted forward on the humerus to perform motion 1. The elbow joint was bent to perform motion 2, the humerus was rotated around the *Y*-axis to perform motion 3, and the wrist joint was inwardly bent to perform motion 4. Finally, experimental motion returned to its initial state and repeated the next set of cycles. Two sets of identical experimental motions were used.

The coordinates of the six marker points were obtained from the motion capture system, and the raw data were smoothed using the sliding average filtering algorithm. The calculated values were estimated using P-BTBS. The exact values were acquired from the motion capture system and smoothed using a sliding average filtering algorithm. The motion capture system and P-BTBS differently defined rotation and the values could be opposite; therefore, the exact values were taken as the opposite. In accordance with the simplified model of humeral motion, the *θ*_5_ of humerus was not significant in the second category, which does not help determine humeral posture. The angular variation of the remaining six DOFs is shown in Fig. 7b–g.

Root Mean Square Error (RMSE) was used as the error evaluation method in this study, which is widely used in engineering measurements. In practical applications, RMSE is sensitive to the data and measures the deviation between the measured value and the true value, thus reflecting the precision of the measurement method.

The experimental motions were executed twice, and all data had two cycles. The shoulder complex was moved passively throughout the experiment; therefore, the two rotation angles of the clavicle changed minimally. The motion angle *θ*_1_ of the clavicle ranged from 0.8733° to 8.9923° with a RMSE of 1.3877°. A forward arm extension was included in the experimental motions, and the *θ*_2_ of clavicle varied more than *θ*_1_. The motion angle *θ*_2_ of the clavicle ranged from − 12.5627° to 1.4329° with an RMSE of 1.0119°. The motion angle *θ*_3_ of the humerus ranged from 1.9542° to 38.7424° with an RMSE of 2.0933°. In its initial state, the humerus was lifted forward; therefore, the motion angle *θ*_4_ of the humerus ranged from 36.2078° to 85.1273° with an RMSE of 2.2824°. The motion angle *θ*_6_ of the forearm ranged from 2.5508° to 89.7897° with an RMSE of 0.9814°. The motion angle *θ*_7_ of the hand ranged from − 51.3814° to 7.6649° with an RMSE of 1.8875°.

The exact values obtained from the motion capture system were the gold standard compared with the P-BTBS results and the mean value of RMSE was 1.6074°. Moreover, compared with the traditional vector method, P-BTBS estimated three to eight motion angles with one coordinate transformation, which can reduce the errors caused by multiple transformations.

More importantly, a maximum of six spatial points was required to estimate the eight motion angles of the upper limb. The locations of the six spatial points coincided with those of Kinect's human skeleton recognition technology^33^, which encourages the application of P-BTBS to most scenarios for estimating the spatial coordinates.

### Description of upper-limb motions

Upper-limb motions are complex, unordered, and subconscious. Explaining the subconscious actuation of brain such as picking up objects from a table is challenging. P-BTBS implements the function of describing upper-limb motions through mathematical methods, which provides a way to observe upper-limb motions. P-BTBS constructs a mathematical framework for multi-space fusion and derives models (Eqs. (19) and (23)) for describing upper limb motions in Euclidean space (*E*^O^) and TPS (*E*^T^), respectively. This mathematical framework not only enriches the motion information mathematically, but more importantly reflects the real upper limb motions, which makes it possible to accurately describe these complex motions of the upper limb.

**Figure 8:**
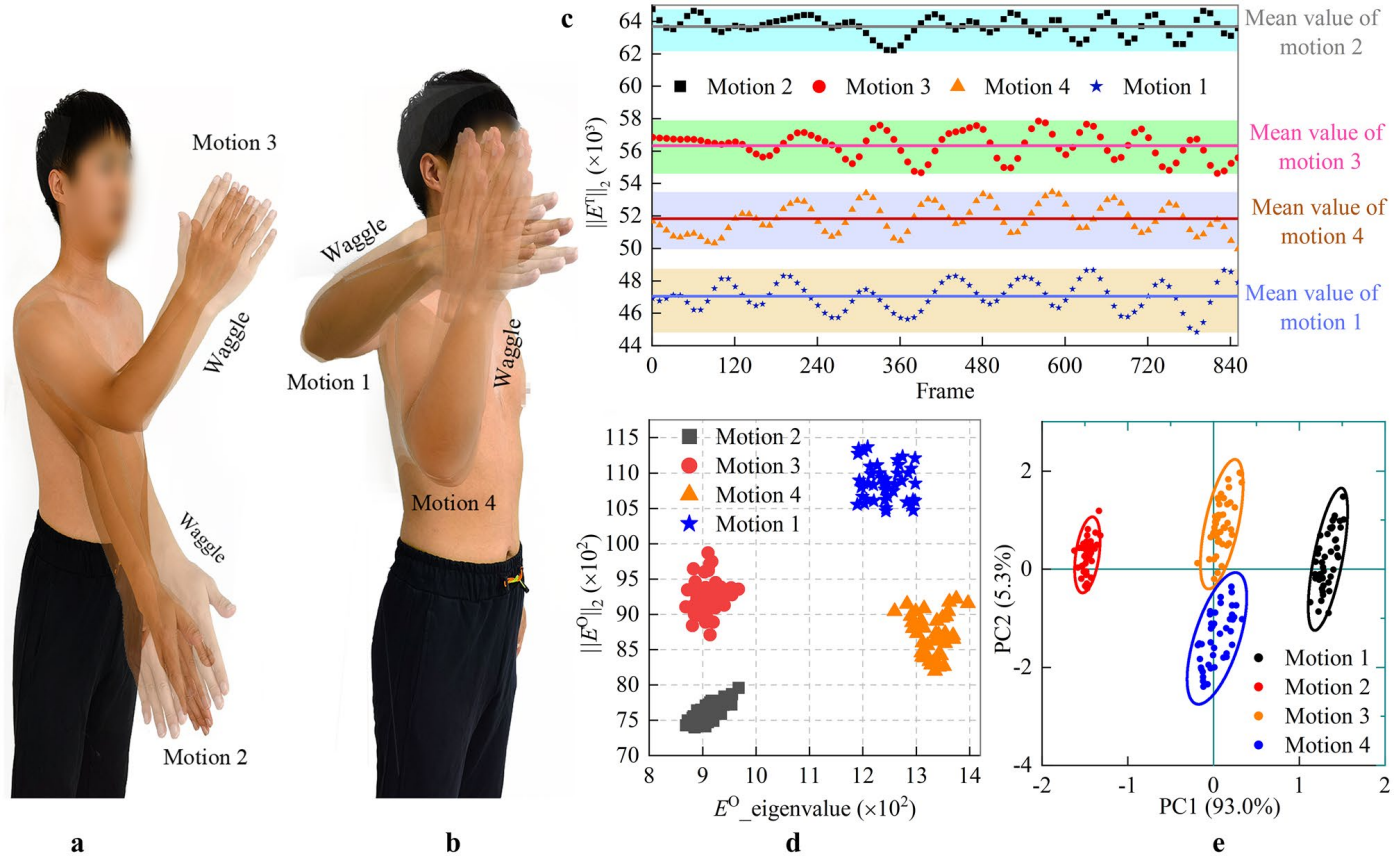
The experiment of upper-limb motions description. (**a**,**b**) The four groups of motions are described. Each set of motions is performed with the same waggle amplitude. (**c**) The 2-Norm of *E*^T^ matrices obtained in TPS is calculated for each frame, with each group of values having a cyclical variation around its mean value, and having a visible difference in the mean values. Just one point per ten data points is displayed, the rest are skipped. (**d**) The 2-Norm and eigenvalues’ mean value of *E*^O^ matrix in Euclidean space are calculated for each frame to form a 2D planar map. The points representing the different motions appear in different regions of the 2D plane. Just one point per twenty data points is displayed, the rest are skipped. (**e**) Principal component analysis (PCA) and data downscaling are performed on the three-dimensional features of each frame, with the three-dimensional features being the 2-Norm of *E*^O^, the eigenvalues’ mean value of *E*^O^ and the 2-Norm of *E*^T^, respectively. Just one point per twenty data points is displayed, the rest are skipped.

In the motion description experiments, the participants and experimental field layout were the same as those in the joint angle-estimation experiments. Four motions with the same wiggling amplitude were performed, as shown in Fig. 8a and b. The experimental procedure is shown in Supplementary Video 2. Fixed motions were not chosen because the high contingencies were not sufficient to demonstrate the ability of the P-BTBS to describe upper limb motions. Dynamic wiggling motions are more in line with real-world upper limb motions because of the dynamic nature of the motions in the real world. The four sets of experimental motions were designed because they encompassed the range of upper limb motions (front and side of the body) in everyday life.

The *E*^O^ and *E*^T^ of each frame were calculated in Euclidean space and TPS, respectively, whereas the features were analyzed only in Euclidean space. The independent variables in *E*^T^ (i.e. *M*, *N*, and *U*) have physical significance only in TPS. Thus, *E*^O^ and *E*^T^ are numerical matrices of dimensions 4 × 4 and 6 × 6 in Euclidean space, respectively.

When the one-dimensional feature of P-BTBS was utilized to describe the motions, the 2-Norm of *E*^T^ was calculated. The variation with 2-Norm characteristics of the four motions is shown in Fig. 8c. The 2-Norm of *E*^T^ for each motion has a cyclical variation around its mean value.

When the two-dimensional feature of P-BTBS was utilized to describe the motions, the 2-Norm and eigenvalue mean of *E*^O^ values were calculated. Each *E*^O^ has four eigenvalues, which can be complex numbers and exist in pairs. Thus, the mean value of the four eigenvalues remains a real number. The mean values of the eigenvalues of *E*^O^ and 2-Norm were utilized as the *X* and *Y* axes, respectively, as shown in Fig. 8d. A wiggling motion can be clustered in a region.

To verify the accuracy of *E*^O^ and *E*^T^ in describing upper-limb motions, a three-dimensional (3D) dimension reduction analysis was performed. The 2-Norm of* E*^O^, mean of eigenvalues of *E*^O^, and 2-Norm of *E*^T^ construct a 3D feature for each frame. Principal components (PC) 1 and PC 2 are shown in the *X* and *Y* axes of the principal component analysis (PCA) diagram, respectively, with PC1 at 93% and PC2 at 5.3%, as shown in Fig. 8e.

One point in the PCA diagram corresponds to the motion frame. The four sets of wiggling motions corresponded to the four 95% confidence ellipses. The confidence ellipses of motions 3 and 4 were close but did not intersect. This is because motions 3 and 4 are similar, but fundamentally different (fixed relative posture of the humerus and forearm, only humerus rotates around the *Y*-axis), reflecting both the differences and similarities in upper-limb motions.

In conclusion, P-BTBS can classify and cluster upper-limb motions utilizing one-dimensional features (Fig. 8c), two-dimensional features (Fig. 8d), and 3D dimension reduction features (Fig. 8e), which demonstrates its effectiveness.

## Discussion

The above modeling process and experimental results demonstrate that the proposed P-BTBS conforms to the physiology^66^ of human upper limbs, possesses high accuracy of motion angles (mean value of RMSE is 1.6074°), and describes upper-limb motions with high accuracy. Defining generic upper-limb spatial points extends the application scenario of P-BTBS, which satisfies the generic nature of equipment and technology. Modular motion of the upper limb by constructing multi-space-fusion, which satisfies the generic nature of study targets (1–8 DOFs) in accordance with research topics. Moreover, the upper limb is not a continuous kinematics chain due to the presence of the scapula, and P-BTBScan handle this type of issue. In practical applications, the P-BTBS primary coordinate system transformation eliminates the step of defining multiple coordinate systems (compared to the Denavit-Hartenberg model), reduces the impact of cumulative errors on results (compared to the spatial vector method), simplifies the application process, and extends the range of applicability.

P-BTBS provides a new perspective and tool for understanding and exploring upper-limb motions, which can be utilized in a wide range of fields. Specifically, in the following three fields:

1. Upper-limb rehabilitation^8–10^. P-BTBS can provide rehabilitation robots with information on patients’ motions (for example, mirror rehabilitation)^21,54^ and physician guidance (such as passive intervention rehabilitation). The information organically integrates the upper-limb rehabilitation robot with the patient's autonomous consciousness, which can improve rehabilitation efficacy and alleviate the conflict between patients and medical rehabilitation. Among existing methods, motion capture systems cannot be widely promoted; multi-camera data fusion, inertial sensors, and wearable devices offer limited DOFs of the upper limb (less than 6 DOFs).
2. Assessment of upper-limb motor function^55,56^. The P-BTBS can extract upper-limb motion angles and analyze the accuracy of reaching motions, which can be utilized to assess upper-limb motor function. Timely identification of the rehabilitation stage is essential for patients with upper-limb motion disorders due to stroke^67^. Physicians can accurately grasp patients’ rehabilitation stages using P-BTBS and then develop timely rehabilitation strategies. Among existing methods, the assessment of upper limb motor function lacks diversity (only upper limb motion angles can be assessed, or only motion-reaching ability can be assessed).
3. Motion recognition and classification. Motion recognition and classification have been important research directions^68,69^. Making the computer “see” or “sense” upper-limb motions is an important part of program execution or function implementation. P-BTBS provides a new mathematical tool for computers to observe human upper-limb motions without training redundant neural networks that utilize abundant samples. Existing methods typically use various neural network models to train images, depth images, biosignals, etc., which is a complicated process with many interfering factors and requires abundant samples.

Upper-limb motions involve both rotation of the joints and a small sliding motion of the plane joints (such as the acromioclavicular joint). The scapula maintains biomechanical stability and provides the drive. However, the small sliding motion and scapula were not considered in P-BTBS. The sliding motion was not considered because even sliding motion can improve upper-limb flexibility to some extent^66^; there is no clear evidence that sliding motion has an absolute effect on upper-limb motions. The scapula is enveloped by skin and muscle tissue, making its observation with non-invasive methods difficult. Thus, the scapula was simplified as a passively moving bone in P-BTBS. In addition, the P-BTBS analyses a maximum of 8 DOFs, which is a potential limitation in some application scenarios where high DOFs (greater than 8 DOFs) are required.

P-BTBS provides an ingenious and convenient solution for analyzing and extracting human upper-limb motion information, which can be used by most equipment for estimating spatial coordinates. The extraction and description of upper-limb motion information can provide theoretical references for practical engineering applications. Moreover, combined with Internet of Things technology, P-BTBS has promising potential for applications, such as upper-limb rehabilitation, motor function assessment, computerized recognition and classification, behavioral monitoring, senior care, human–robot interaction, and robot control strategies.

In the future, P-BTBS will be committed to promoting engineering applications and experimental explorations. P-BTBS can be integrated into upper-limb rehabilitation robots to conduct upper-limb rehabilitation after stroke, which can improve human–machine interaction, enrich rehabilitation modalities, and increase rehabilitation efficacy (Supplementary Video 1).

## Methods

### Data acquisition and analytics

Eight optical motion capture cameras were used to cover the entire experimental field, and the frame rate was set to 60 Hz. The data were collected and processed offline using a host computer. All raw data were filtered using a sliding average filter algorithm with filter type of moving average and a filter coefficient of 25.

The virtual spatial points *P*_11_ and *P*_12_ held singular positions when line segment *l*_9,10_ was vertically horizontal (ground), resulting in multiple sets of solutions of Eq. (1). In the singular position, the following equation holds, and the coordinates of *P*_11_ and *P*_12_ are selected as (*x*_10_, *y*_10_, *z*_10_ + *L*_10,13_) and (*x*_10_ + *L*_10,13_, *y*_10_, *z*_10_), respectively:

$$ y_{9} - y_{10} = L_{9,10} $$

### Ethics approval and informed consent

All experimental protocols and methods were approved by the Medical Ethics Committee of Harbin Institute of Technology with Ethics No: HIT-2022032 and carried out in accordance with relevant guidelines and regulations. This study confirms that informed consent was obtained from all subjects and/or their legal guardian(s). This study obtained informed consent from all subjects and/or their legal guardian(s) for the publication of identifying information/images in an online open-access publication.

### Supplementary Information

Supplementary Video 1.

Supplementary Video 2.

## Data Availability

The data supporting the findings of this study are available from the corresponding author upon reasonable request.

## Code availability

Codes that support the findings of this study are available from the corresponding author upon reasonable request.
